# Synthesis of Biogenic Silver Nanoparticles (AgCl-NPs) Using a *Pulicaria vulgaris* Gaertn. Aerial Part Extract and Their Application as Antibacterial, Antifungal and Antioxidant Agents

**DOI:** 10.3390/nano10040638

**Published:** 2020-03-29

**Authors:** Majid Sharifi-Rad, Pawel Pohl

**Affiliations:** 1 Department of Range and Watershed Management, Faculty of Water and Soil, University of Zabol, Zabol 98613-35856, Iran; 2 Department of Analytical Chemistry and Chemical Metallurgy, Faculty of Chemistry, University of Science and Technology, Wyspianskiego 27, 50370 Wroclaw, Poland

**Keywords:** biomedical application, green synthesis, silver chloride nanoparticle, *Pulicaria vulgaris*

## Abstract

In this study, very simple and fast one-step synthesis of biogenic silver chloride nanoparticles (AgCl-NPs) using a *Pulicaria vulgaris* Gaertn. aerial part extract from an aqueous solution of silver nitrate at room temperature is proposed. The proceedings of the reaction were investigated by UV–Vis spectroscopy. AgCl-NPs were characterized using X-ray diffraction spectroscopy (XRD), Fourier-transform infrared spectroscopy (FTIR), and transmission electron microscopy (TEM). Antibacterial and antifungal activities of these nanoparticles were evaluated by disk diffusion and microdilution methods against *Staphylococcus aureus*, *Escherichia coli*, *Candida albicans,* and *C. glabrata*. In addition, the antioxidant activity of the synthesized AgCl-NPs was determined by the DPPH radical scavenging assay. The antimicrobial test confirmed the bactericidal activity of biosynthesized AgCl-NPs against Gram-positive and Gram-negative bacteria. They also exhibited good antifungal activities with minimum inhibitory concentration (MIC) values ranging from 40 to 60 µg/mL against *Candida glabrata* and *Candida albicans*, respectively. In addition, biosynthesized AgCl-NPs were established to have remarkable antioxidant activity. All this pointed out that the proposed new biosynthesis approach resulted in production of AgCl-NPs with convenient biomedical applications.

## 1. Introduction

Nanotechnology is the most fascinating field of study in modern material sciences [1]. Nanoparticles (NPs) show a particle size ranging from 1 to 100 nanometers (nm) [2]. Because of the large surface area to volume ratios, nanoscale materials have unique and superior physicochemical characteristics in comparison to their bulk structures [3]. Most of studied NPs are metallic because their syntheses are easier. In addition, monometallic or bimetallic NPs have a wide range of applications; i.e., from detectors, chemical sensors, and catalysts, to surface coating agents, electronic components, photographic components, and pharmaceutical products [4,5]. Metallic NPs can be synthesized using different methods; i.e., chemical, physical, and biological. Conventional physical and chemical methods normally include expensive physical and chemical processes that often use substrates or are accompanied by byproducts, which pose cytotoxicity, environmental toxicity, and carcinogenicity [6]. Bio-based synthesis, facilitated by the use of enzymes, microorganisms, and cell or plant extracts to reduce dissolved metal ions, is considered as an inexpensive, simple, nontoxic, and environment-friendly approach to the production of well characterized, highly stable, bio-compatible, and safer metallic NPs [7,8]. Some plant extracts contain secondary metabolites that act as bio-reductants, stabilizers, or both, in the process of NPs formation [9,10]. Silver/silver chloride NPs (Ag/AgCl-NPs) have gained considerable attention, especially in the fields of medicine, pharmacology, and health protection [11]. AgNPs are well recognized to exhibit antibacterial and antifungal properties, and pose antioxidant activity [12,13]. Reduced Ag is known to affect a wide range of biological processes occurring in microorganisms, and is involved in their proper functioning and development, and the structures of their cell membranes. This is due to interactions between Ag(I) ions and different macromolecules present in these cells. For instance, Ag(I) ions, blocking synthesis of proteins, reduce in turn, membrane permeability, and ultimately lead to cell death [14,15]. AgNPs have stronger biological activity in comparison to bulk Ag; hence, they are more effective and reactive [16].

The genus *Pulicaria* is a member of the Asteraceae family, which includes approximately 100 species; mainly distributed in the North Africa, Europe, and Asia, as well as the Mediterranean region [17]. This genus is represented in the Iranian flora by five species [18]. Phytochemical studies on *Pulicaria* species showed that they are rich in sesquiterpenes, monoterpenes, triterpenes, diterpenes, steroids, flavonoids, and phenolic compounds [17]. Therefore, they are commonly used in folk medicine to treat chills, cardiac disorders, diabetes, inflammation, abscesses, and skin diseases [19]. They can also be used as natural insect repellents [19]. Different biological activities on living organisms of *Pulicaria* species, such as cytotoxicity [20], along with antibacterial [21] antioxidant [22], and antifungal [23] properties, have been reported so far.

Due to importance of synthesis of biogenic AgNPs that could have certain biological activities and could be potentially applied in medicine, the aim of this study was to develop the new green synthesis procedure of AgCl-NPs using a *Pulicaria vulgaris* Gaertn. aerial part extract. Antibacterial, antifungal, and antioxidant properties of the resulting nanomaterial were evaluated. To the best of our knowledge, this is the first report on synthesis of biogenic AgCl-NPs using the extract of the aerial part of this pharmaceutical plant.

## 2. Materials and Methods

### 2.1. Plant Material

The material of the *Pulicaria vulgaris* Gaertn. plant was collected during the flowering stage in September 2019—from Saravan rangelands, Sistan and Baluchistan Province, Iran—including the flowers, leaves, and stems of this plant. The voucher specimen (number 12832) was deposited in the herbarium, Department of Biology, University of Zabol, Zabol, Iran. Aerial parts of collected plants were air dried, and ground to a fine powder by an electric grinder (Pars khazar, Tehran, Iran). The plant extract was prepared by treating 2.5 g of the dried and powdered plant material (aerial parts of *Pulicaria vulgaris* Gaertn.) with 50 mL of ethanol (98%) for 24 h at room temperature and under magnetic stirring. Resulting mixtures were filtered through a Whatman filter paper number 1 to separate the extract from the spent plant material. The filtrate was directly applied for synthesis of AgCl-NPs.

### 2.2. Green Synthesis of AgCl-NPs

For the green synthesis of AgCl-NPs, to 1 mmol/L AgNO_3_ (Merck, Darmstadt, Germany) solutions the plant extract was added so that its final concentration in resultant mixtures was 1%, 2%, 4%, and 6% (v/v). These mixtures were allowed to react at room temperature for 210 min. After that, resulting AgCl-NPs were gathered via centrifugation (18,000 g, 20 min). Pellets formed were washed three times by sterile distilled water, and then dried at room temperature. Dried biogenic AgCl-NPs were stored in microtubes for further analysis.

### 2.3. Characterization of AgCl-NPs

AgCl-NPs’ formation during their biological synthesis was evaluated using UV–Vis absorption spectroscopy. Reaction mixtures were sampled at various times, and their absorption spectra in the range of 200–800 nm were acquired with an UV–Vis spectrophotometer (UV-1800 240 V, Shimadzu Corporation, Kyoto, Japan).

The presence of a variety of functional groups from the plant extract, which could contribute to synthesis of biogenic AgCl-NPs, was evaluated by FTIR spectroscopy. A mixture of dried AgCl-NPs and KBr was ground into a fine powder and pressed into a pellet. Using a Nicolet 800 FTIR spectrometer (Nicolet, Madison, USA), absorption spectra of AgCl-NPs included in pellets were acquired at wavelengths ranging from 4000 to 400 cm^−1^ at resolution of 4 cm^−1^, as described by Das et al. [24].

Crystalline behavior of synthesized biogenic AgCl-NPs was evaluated by X-ray diffraction (XRD), using a Cu-K*α* radiation source (*λ* = 1.54 Å). Diffractograms were scanned in the region of 2*θ* from 10° to 80° at a rate of 0.026°/min, using an X-ray diffractometer (Siemens D5000, Munich, Germany). The Debye–Scherer equation was applied to determine the average crystallite size of biogenic AgCl-NPs, as described by Bagherzade et al. [25].

The average sizes and shapes of synthesized biogenic AgCl-NPs were assessed using a Philips GM-30 transmission electron microscope (Hillsboro, OR, USA), operated at 120 kV with 2.5 Å resolution, as recommended by a TEM instrument supplier. One drop of an AgCl-NP-containing solution was put onto a Cu grid and evaporated to dryness under an infrared lamp. Digimizer software (version 4.1.1.0, MedCalc Software, Ostend, Belgium) was used for determination of particles size distribution based on TEM images.

### 2.4. Antibacterial and Antifungal Activities

#### 2.4.1. Microorganisms

Biogenic AgCl-NPs were tested against 4 microorganisms, including *Staphylococcus aureus* (ATCC 29737), *Escherichia coli* (ATCC 10536), *Candida albicans* (ATCC 10231), and *Candida glabrata* (ATCC 90030). All microorganisms were obtained from Iranian Research Organization for Science and Technology (IROST). Bacterial and fungal strains were incubated overnight at 37 °C on a nutrient broth and a Sabouraud dextrose agar (Merck KGaA, Darmstadt, Germany) containing 5% chloramphenicol [26].

#### 2.4.2. Disc Diffusion Method

Antimicrobial and antifungal activities of AgCl-NPs were evaluated using the disc diffusion method as described by National Committee for Clinical Laboratory Standards (NCCLS) [26]. Accordingly, 100 μL of 0.5 McFarland standard suspensions of tested microorganisms, which contained 1.5 × 10^8^ CFU/mL of bacteria or 1.5 × 10^6^ CFU/mL of fungal strains, were applied in these tests. Mueller–Hinton (Merck KGaA, Darmstadt, Germany) and Sabouraud dextrose agars were used to inoculate tested bacterial and fungal strains. Different concentrations of biogenic AgCl-NPs (20 and 40 µg/mL), dispersed in sterile distill water, were used in these experiments. Solutions of amoxicillin (20 and 40 µg/mL) and fluconazole (20 and 40 µg/mL), both purchased from Farabi pharmaceutical Company, Iran, were also used in combination with AgCl-NPs for antibacterial and antifungal activity, respectively. Sterilized paper discs (6 mm in diameter) were impregnated with 10 μL of above mentioned solutions, and allowed to dry at room temperature. After that, disks were placed on Petri dishes, and inoculated by tested microorganisms. Discs treated with DMSO were used as negative controls. All Petri dishes were incubated at 37 °C for 24 h (bacterial strains) or 48 h (fungal strains). Susceptibility of the tested microorganisms to synthesized biogenic AgCl-NPs was evaluated by measuring appropriate diameters of resulting inhibition zones (in mm).

#### 2.4.3. Determination of Minimum Inhibitory Concentration (MIC) 

The minimum inhibitory concentration (MIC) was measured by using the microdilution assay, as explained by the Clinical and Laboratory Standards Institute (CLSI) [27]. In case of antibacterial activity, serial dilutions of a AgCl-NP bulk solution (at 20, 40, 60, 80, 100, and 120 µg/mL) were made in a 96-well microtiter plate, using the Muller–Hinton broth medium. To evaluate antifungal activity, serial dilutions of the above mentioned AgCl-NP solution (at 20, 40, 60, 80, 100, and 120 µg/mL) were made, using the RPMI-1640 medium (Merck KGaA, Darmstadt, Germany) supplemented with 3-morpholinopropane-1-sulfonic acid (MOPS) (Sigma, St. Louis, MO, USA). In total, 100 μL of 0.5 McFarland standard suspensions of the aforementioned microorganisms were then added to wells of microtiter plates and incubated at 37°C for 24 or 48 h for bacterial and fungal strains, respectively. Media with inoculums but without AgCl-NPs were intended as negative controls, while amoxicillin and fluconazole were used as positive controls for bacteria and fungi, respectively. MIC values reflected the lowest concentrations of biogenic AgCl-NPs that led to no visible growth of the microorganisms.

#### 2.4.4. Minimum Bactericidal Concentration (MBC) and Minimum Fungicidal Concentration (MFC)

The minimum bactericidal concentration (MBC) and the minimum fungicidal concentration (MFC) were assessed according to CLSI [27]. In short, 100 μL aliquots of media from wells containing bacteria and fungi, in which growth was not visible, were sub-cultured on Mueller–Hinton agar and Sabouraud dextrose agar plates. These plates were incubated for 24 h at 37 °C. The lowest concentrations of AgCl-NPs that showed no bacterial and fungal growth were considered as MBC and MFC values, respectively. 

### 2.5. Antioxidant Properties of the Biosynthesized AgCl-NPs

#### DPPH Radical Scavenging Activity

2,2-diphenyl-1-picrylhydrazyl (DPPH) from Merck (Merck KGaA, Darmstadt, Germany) was used to measure free radical scavenging potential of biosynthesized AgCl-NPs, as described by Brand-Williams et al. [28] with slight modifications. Methanolic solutions of AgCl-NPs at various concentrations (20, 40, 60, 80, 100, and 120 µg/mL) were prepared. Methanolic solutions of butylated hydroxytoluene (BHT) at the same concentrations were also prepared and used as standards. One milliliter aliquots of the abovementioned solutions were added to 2 mL of a methanolic solution of DPPH (1 mmol/L), and thoroughly vortexed. Resulting mixtures were incubated in dark for 30 min at room temperature. After that, the absorbance value of each mixture was measured at 517 nm. The DPPH absorbance value was considered as a control. Free radical scavenging activity of AgCl-NPs or BHT solutions was expressed as the inhibition percentage that was calculated by the following equation:

DPPH radical scavenging activity (%) = 100 × (*A_c_*−*A_s_*)/*A_c_*, where A_c_ is absorbance of the control (containing solvent and DPPH), *A_s_*—absorbance measured for solutions of AgCl-NPs or BHT.

### 2.6. Statistical Analysis

Data were analyzed using the statistical software package SPSS version 11.5 (IBM Corporation, Armonk, NY, USA). The one-way analysis of variance test (ANOVA) and the Duncan’s multiple range test were applied to investigate differences between various groups. All results were expressed as mean values ± standard deviations (SDs).

## 3. Results and Discussion

### 3.1. Visual Confirmation of Green Synthesis of AgCl-NPs

**Figure 1:**
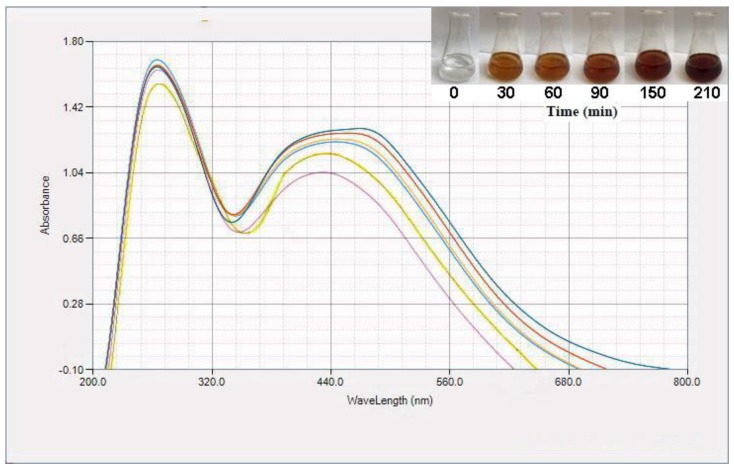
UV–Vis spectra of biosynthesized AgCl- NPs using aerial part extracts of the *Pulicaria vulgaris* plant at various times (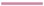
15, 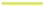
30, 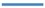
60, 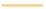
90, 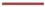
150 and 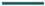
210 min). Additionally, color changes of reaction mixtures are shown.

Differently concentrated (1%, 2%, 4%, and 6%) ethanolic extracts of aerial parts of *P. vulgaris* were used for synthesis of biogenic AgCl-NPs. It was established that only in case of the addition of the 6% (v/v) extract to a 1 mmol/L AgNO_3_ solution, would bioreduction and biosynthesis processes take place, and the colorless AgNO_3_ solution became dark brown with time (Figure 1). This is likely attributable to the completion of the synthesis process, and the formation of biogenic AgCl-NPs [11]. It was previously reported that *P. vulgaris* contains alkaloids, tannins, flavonoids, and phenolic compounds [29] that could be responsible for the bioreduction of Ag(I) ions into AgCl-NPs as well as their capping in the solution [30]. The green synthesis mechanism of AgCl-NPs is not clear yet, but it seems that secondary metabolites of *P. vulgaris* played an important role in their formation and stabilization. These compounds, containing hydroxyl and ketonic groups, likely bound Ag(I) ions—which was necessary to reduce them to form appropriate nuclei [31]. During the development phase, AgCl nuclei were grown to form spherical AgCl-NPs, which were additionally stabilized by mentioned plant metabolites [31].

### 3.2. Characterization of Biosynthesized AgCl-NPs

#### 3.2.1. UV–Visible Spectroscopy

UV–Vis spectra of resulting reaction mixtures were measured at 15, 30, 60, 90, 150, and 210 min after mixing the AgNO_3_ solution and the ethanolic 6% (v/v) plant extract (Figure 1). According to these spectra, intensity of the localized surface plasmon resonance (LSPR) absorption band was increased with time and its maximum was observed at approximately 460 nm, which corresponded to formation of spherical AgCl-NPs [32,33]. UV–VIS spectra of reaction mixtures were also acquired at times higher than 210 min. These spectra overlapped with the spectra acquired at 210 min that pointed out that the end of reaction was reached.

#### 3.2.2. FTIR Spectroscopy

**Figure 2:**
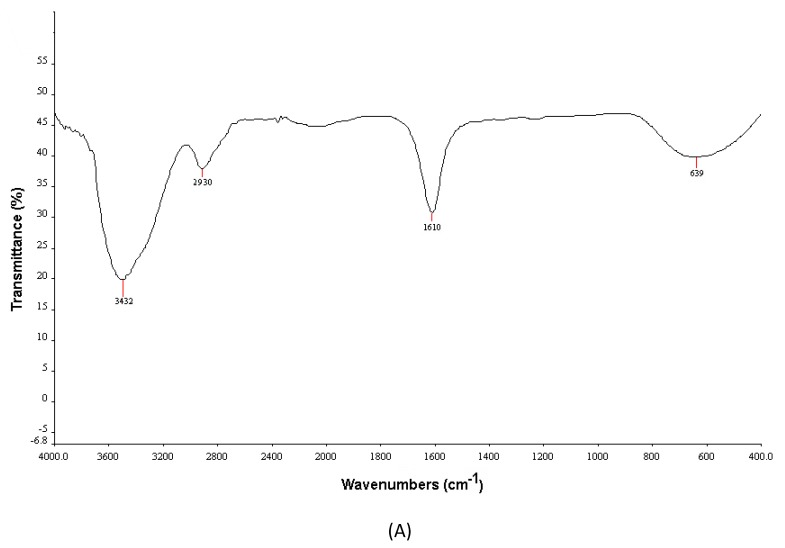

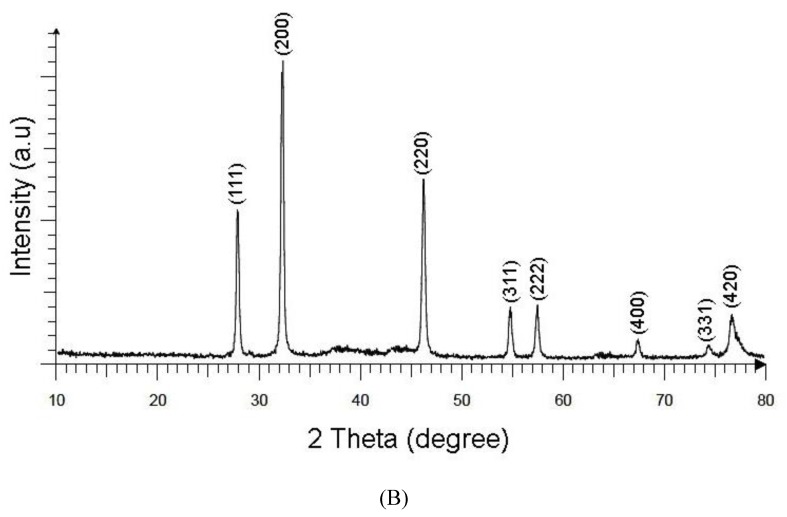
(**A**) The FTIR spectrum and (**B**) the XRD pattern of biosynthesized AgCl-NPs using aerial part extracts of the *P. vulgaris* plant.

FTIR analysis was carried out to identify groups of compounds present in the extract that were linked to biosynthesized nanoparticles (Figure 2A). C–Cl stretching vibrations were observed at 639 cm^−1^ and corresponded to alkyl halides [34]. Absorption bands at 1610 cm^−1^ were assigned to C=C and carbonyl (C=O) stretching vibrations of amide groups (amide I/II), relating to functional groups of proteins and peptides, deformations of aromatic rings, and C=O stretching vibrations related to flavonoids [35]. C–H stretching vibrations were found at 2930 cm^−1^, and were characteristic of aromatic components [25]. The absorption band at 3432 cm^−1^ corresponded to OH functional groups in phenolic components and alcohols with strong hydrogen bonds [25]. All these FTIR absorption peaks verified that synthesized AgCl-NPs were capped with *P. vulgaris* metabolites, likely including phenolic compounds and flavonoids. These compounds stabilized their structure and likely added novel properties to biosynthesized nanoparticles.

#### 3.2.3. X-ray Diffraction (XRD)

Crystalline properties of biosynthesized nanoparticles were evaluated using XRD (Figure 2B). Main diffraction peaks identified in the diffractogram were at 2*θ* values of 27.9°, 32.4°, 46.2°, 54.8°, 57.6°, 67.5°, 74.3°, and 76.9^o^. They corresponded to (111), (200), (220), (311), (222), (400), (331), and (420) planes, respectively, and confirmed the face-centered cubic (FCC) structure of AgCl according to the database of Joint Committee on Powder Diffraction Standards (JCPDS), file number 31-1238. XRD analysis confirmed that biogenic AgCl-NPs were synthesized [11]. According to the Debye–Sherrer equation, the average crystalline size of biosynthesized AgCl-NPs was 28.6 nm.

#### 3.2.4. Transmission Electron Microscopy (TEM)

**Figure 3:**
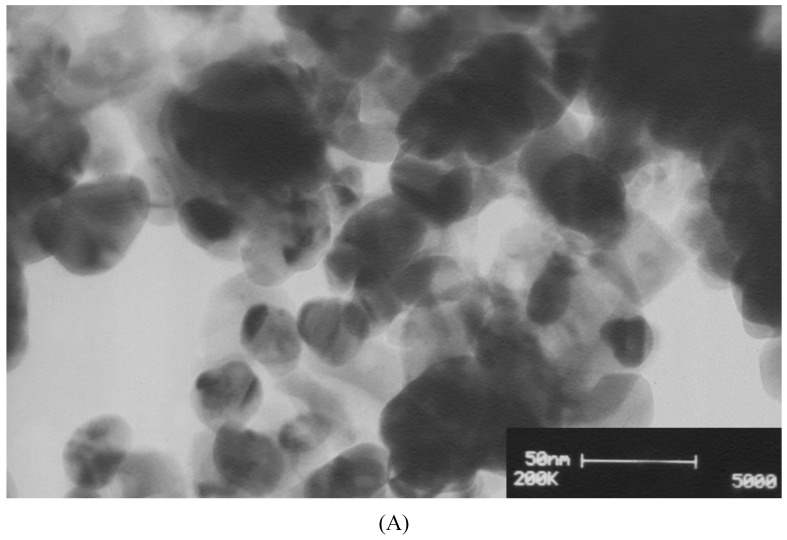

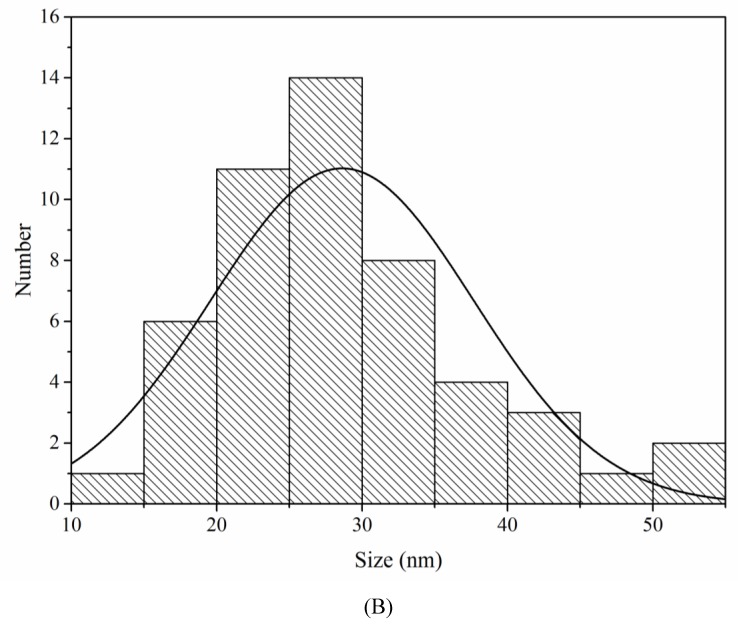
(**A**) The TEM image, and (**B**) the particle size distribution of biosynthesized AgCl-NPs using aerial part extracts of the *P. vulgaris* plant obtained based on TEM images (number of particles = 50). Each bar represents the number of nanoparticles having a size within the particular size range.

TEM analysis (Figure 3A,B) clearly confirmed that the shape of biosynthesized AgCl-NPs was spherical, while their size was ranged from 14.3 to 50.7 nm with the average of 28.6 ± 9.0 nm—that being the same as the particle size measured based on the XRD pattern.

### 3.3. Antibacterial and Antifungal Activities

#### 3.3.1. Disc Diffusion Method

**Figure 4:**
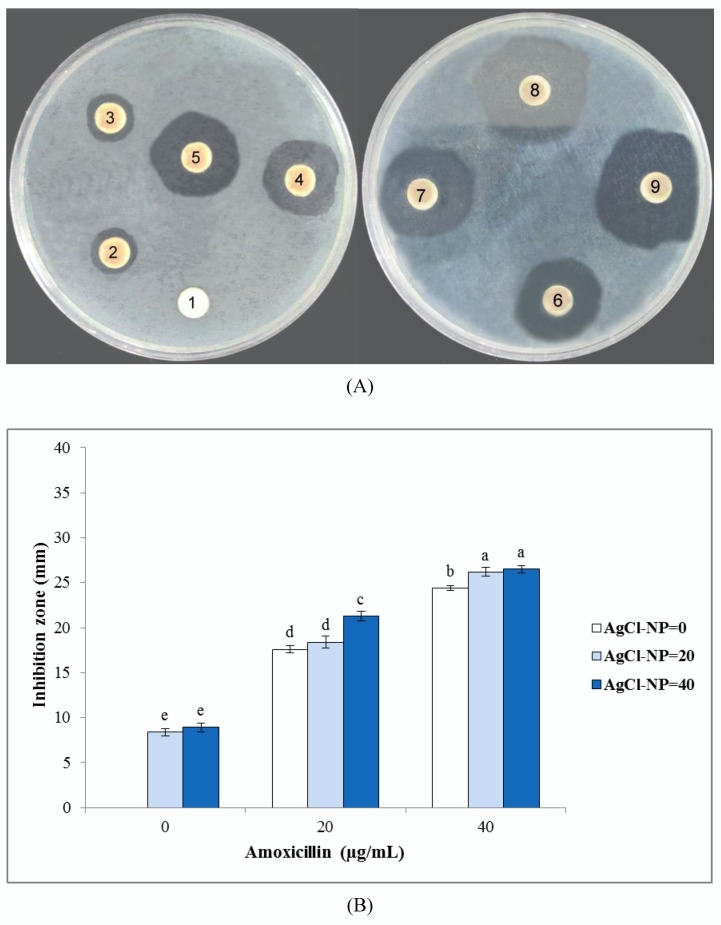
Antibacterial activities of biosynthesized AgCl-NPs against *Escherichia coli*. (**A**) Disk diffusion assay. Different disc numbers showed various concentrations of AgCl-NPs and amoxicillin, as explained in the Material and Methods section (1: control, 2: 20 µg/mL of AgCl-NPs, 3: 40 µg/mL of AgCl-NPs, 4: 20 µg/mL of amoxicillin, 5: 20 µg/mL of AgCl-NPs + 20 µg/mL of amoxicillin, 6: 40 µg/mL of AgCl-NPs + 20 µg/mL of amoxicillin, 7: 40 µg/mL of amoxicillin, 8: 20 µg/mL of AgCl-NPs + 40 µg/mL of amoxicillin, 9: 40 µg/mL of AgCl-NPs + 40 µg/mL of amoxicillin). (**B**) Inhibition zone diameter. Different letters indicate significant differences (*p* < 0.05) among various treatments.

**Figure 5:**
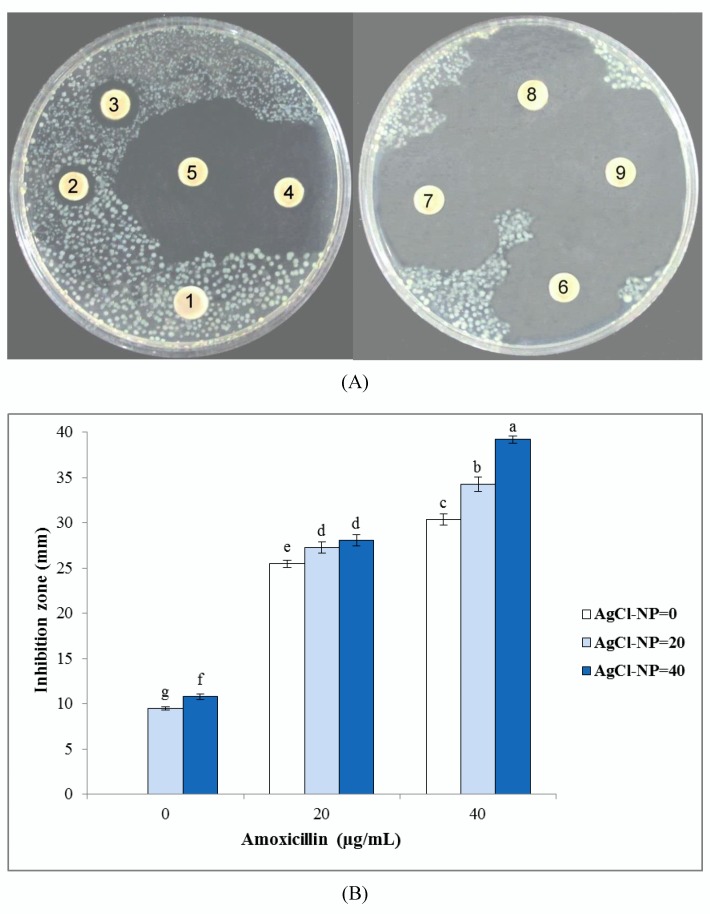
Antibacterial activities of biosynthesized AgCl-NPs against *Staphylococcus aureus*. (**A**) Disk diffusion assay. Different disc numbers showed various concentrations of AgCl-NPs and amoxicillin, as explained in the Material and Methods section (1: control, 2: 20 µg/mL of AgCl-NPs, 3: 40 µg/mL of AgCl-NPs, 4: 20 µg/mL of amoxicillin, 5: 20 µg/mL of AgCl-NPs + 20 µg/mL of amoxicillin, 6: 40 µg/mL of AgCl-NPs + 20 µg/mL of amoxicillin, 7: 40 µg/mL of amoxicillin, 8: 20 µg/mL of AgCl-NPs + 40 µg/mL of amoxicillin, 9: 40 µg/mL of AgCl-NPs + 40 µg/mL of amoxicillin). (**B**) Inhibition zone diameter. Different letters indicate significant differences (*p* < 0.05) among various treatments.

**Figure 6:**
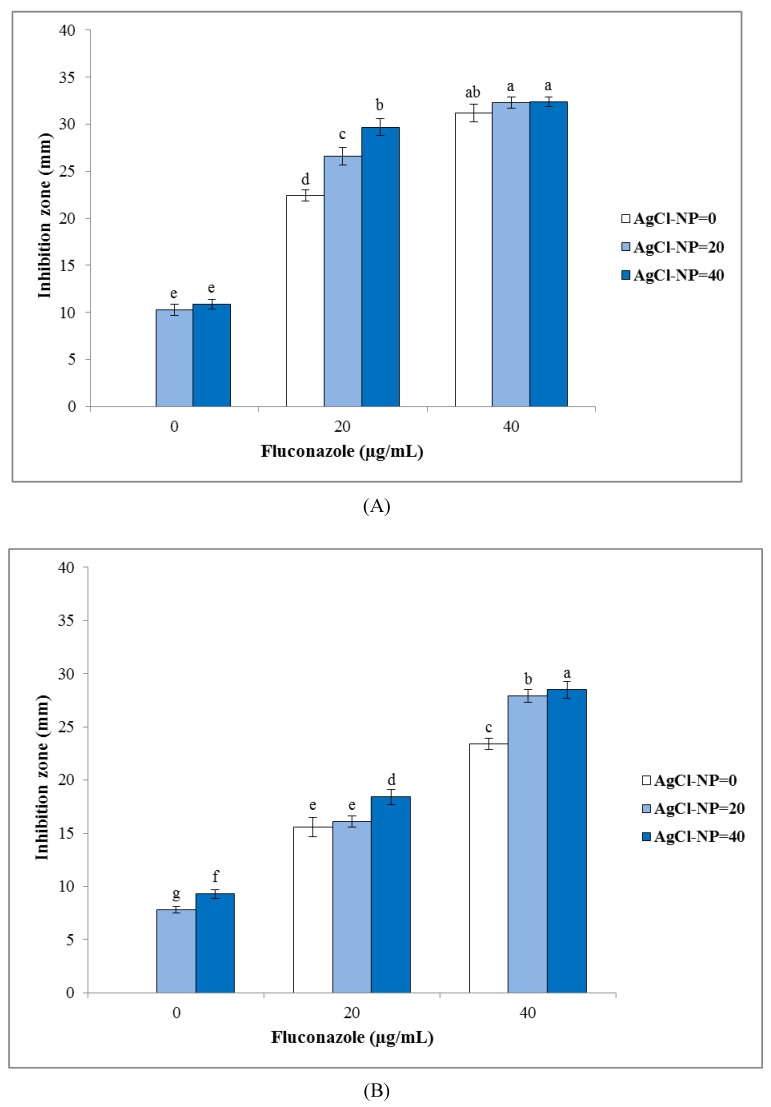
Inhibition zone values of biosynthesized AgCl-NPs alone and in combination with fluconazole against tested fungi strains. (**A**) *Candida glabrata*; (**B**) *C. albicans*. Different letters indicate significant differences (*p* < 0.05) among various treatments.

Results of the inhibition zone test of biosynthesized AgCl-NPs against studied bacterial and fungal pathogens are shown in Figure 4, Figure 5, and Figure 6. They clearly showed that biogenic AgCl-NPs had significant antibacterial and antifungal properties. AgCl-NPs indicated higher inhibition activity against the Gram-positive *S. aureus* strain than against the Gram-negative *E. coli* strain. Greater susceptibility of Gram-positive bacterial strains as compared to Gram-negative bacterial strains to action of AgCl-NPs could be related to differences in membrane permeability of these microorganisms [36]. For all tested bacteria and fungi, 20 or 40 µg/mL solutions of synthesized biogenic AgCl-NPs alone were established to have appropriate antibacterial and antifungal activities. Results obtained in the present work using the disc diffusion method for bacterial and fungal strains could suggest that AgCl-NPs caused physical changes in the structural integrity of the membranes of the studied pathogens, resulting in their permeability. This could be responsible for leakage of cellular contents and cell death, as was also observed in [37,38]. The mechanism of antimicrobial activity of synthesized biogenic AgCl-NPs could also be related to the release of Ag^+^ ions and their effect on studied bacterial and fungal strains [39]. 

Interestingly, a combination of biosynthesized AgCl-NPs with amoxicillin or fluconazole was found to have synergetic effects on their antibacterial and antifungal activities, respectively. Since bacterial cell membranes include hydrophobic structures, they likely prevent the passage of antibiotics. In case of their combination with the AgCl-NP nanostructures used, due to their small particle size, they could enter target cells and enhance the death of pathogens [40]. A similar synergistic effect of biosynthesized AgCl-NPs in combination with antibiotics and fungicides was previously reported by Patra and Baek [13].

#### 3.3.2. Determination of MIC, MBC, and MFC Values

**Figure 7:**
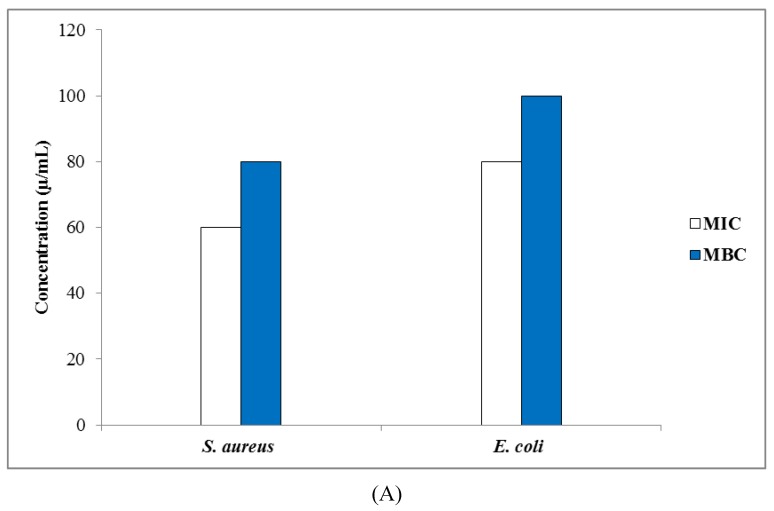

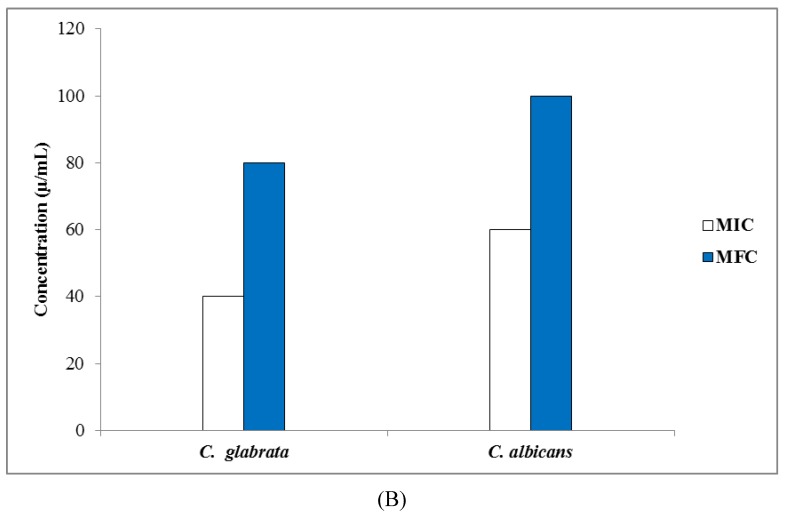
(**A**) Values of minimum inhibitory concentration (MIC) and minimum bactericidal concentration (MBC). (**B**) Values of minimum inhibitory concentration (MIC) and minimum fungicidal concentration (MFC) of biosynthesized AgCl-NPs against tested strains.

Biosynthesized AgCl-NPs showed the best antibacterial activity against all tested bacteria strains with MIC values ranging from 60 to 80 µg/mL (see Figure 7A). MBC values of these AgCl-NPs for *S. aureus* and *E. coli* were 80 and 100 µg/mL, respectively. It was assumed that AgCl-NPs could effectively bind to bacteria biomolecules, and posing high cytotoxicity, limit their activities, and lead to their death [41]. MIC values for tested *C. glabrata and C. albicans* strains were 40–60 µg/mL, respectively. MFC values of these AgCl-NPs were 80 and 100 µg/mL for *C. glabrata* and *C. albicans*, respectively (Figure 7B). These results were in agreement with Salati et al. [42], who investigated biological synthesis of AgNPs by a mango plant extract, and their anti-*Candida* effects. They reported that biogenic AgNPs were most effective against *C. glabrata* as compared to *C. albicans*.

### 3.4. Antioxidant Properties of Biosynthesized AgCl-NPs

**Figure 8:**
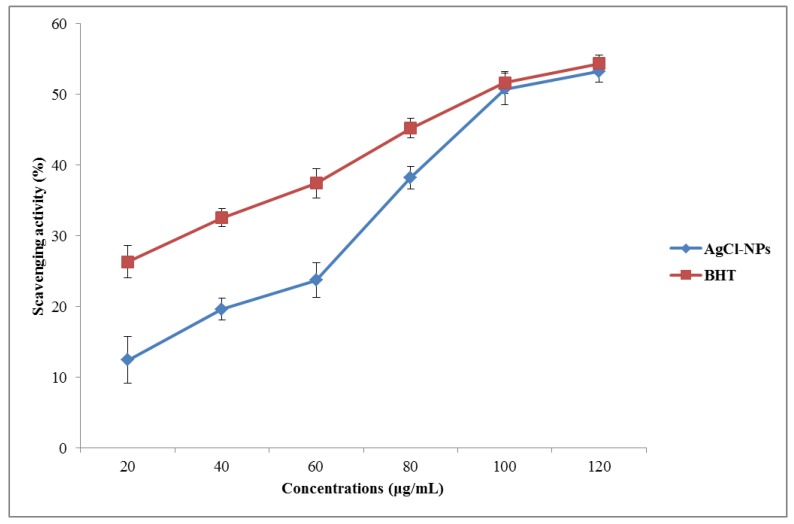
Antioxidant activity of biosynthesized AgCl-NPs from the *Pulicaria vulgaris* aerial part extract.

Free radical scavenging activity of biosynthesized AgCl-NPs was evaluated by the DPPH radical scavenging assay (Figure 8). They had a remarkable scavenging potential, but their radical scavenging was lower when compared to BHT. The observed effect of synthesized biogenic AgCl-NPs on DPPH radicals was likely related to their hydrogen donation activity [43]. It has been reported that the *P. vulgaris* extract contains a high number of polyphenolic compounds [17]. These compounds have strong antioxidants, which help to protect cells from oxidative damage by free radicals. Phenolic compounds entrapped on the surface of AgCl-NPs could enhance their antioxidant activity, having the vital ability to scavenge free radicals and up-regulate certain metal chelation reactions [44]. We suggest that antioxidant properties of AgCl-NPs could be due to the simultaneous activity of AgCl-NPs as a catalyst and polyphenolic compounds on their surfaces as antioxidant agents [45].

## 4. Conclusions

The current study demonstrated the application of ethanolic extracts of *Pulicaria vulgaris* Gaertn. aerial parts for one-stage synthesis of AgCl-NPs in a cost-effective, simple, and environmentally friendly way at room temperature. Results showed that the *P. vulgaris* extract contained organic compounds such that functional groups possibly can act as both reducing and stabilizing agents in the biosynthesis of AgCl-NPs. Biosynthesized AgCl-NPs revealed the LSPR absorption band at 460 nm, and had the average particle size of 28.6 nm, as measured based on TEM images and the XRD pattern. They showed significant antibacterial, antifungal, and antioxidant activates, and had synergetic effects in combination with a conventional antibiotic and a fungicide, amoxicillin and fluconazole, against the tested bacterial and fungal pathogens. These convenient properties of our biogenic AgCl-NPs mean that they could successfully be used as appropriate biocidal agents in biomedical and food industry applications.

## References

[1] Manivasagan P., Kim S.K., Kwon Kim S., Chojnacka K. (2015). Marine Algae Extracts: Processes, Products, and Applications.

[2] Roduner E. (2006). Size matters: Why nanomaterials are different. Chem. Soc. Rev..

[3] Cushing B.L., Kolesnichenko V.L., O’Connor C.J. (2004). Recent advances in the liquid-phase syntheses of inorganic nanoparticles. Chem. Rev..

[4] Shah M., Fawcett D., Sharma S., Tripathy S.K., Poinern G.E.J. (2015). Green synthesis of metallic nanoparticles via biological entities. Materials.

[5] Pareek V., Bhargava A., Gupta R., Navin J., Jitendra P. (2017). Synthesis and applications of noble metal nanoparticles: A review. Adv. Sci. Eng. Med..

[6] Ai J., Biazar E., Jafarpour M., Montazeri M., Majdi A., Aminifard S., Zafari M., Akbari H.R., Rad H.G. (2011). Nanotoxicology and nanoparticle safety in biomedical designs. Int. J. Nanomed..

[7] Gowramma B., Keerthi U., Mokula R., Rao D.M. (2015). Biogenic silver nanoparticles production and characterization from native stain of *Corynebacterium* species and its antimicrobial activity. 3 Biotech.

[8] Gour A., Jain N.K. (2019). Advances in green synthesis of nanoparticles. Artif. Cells Nanomed. Biotechnol..

[9] Jirovetz L., Buchbauer G., Shafi M.P., Leela N.K. (2003). Analysis of the essential oils of the leaves, stems, rhizomes and roots of the medicinal plant *Alpinia galanga* from southern India. Acta. Pharm..

[10] Prakash P., Gnanaprakasam P., Emmanuel R., Arokiyaraj S., Saravanan M. (2013). Green synthesis of silver nanoparticles from leaf extract of *Mimusops elengi*, Linn. for enhanced antibacterial activity against multi drug resistant clinical isolates. Colloids Surf. B Biointerfaces.

[11] Okaiyeto K., Ojemaye M.O., Hoppe H., Mabinya L.V., Okoh A.I. (2019). Phytofabrication of silver/silver chloride nanoparticles using aqueous leaf extract of *Oedera genistifolia*: Characterization and antibacterial potential. Molecules.

[12] Veerasamy R., Xin T.Z., Gunasagaran S., Wei T.F.X., Yang E.F.C., Kumar N.J., Dhanaraj S.A. (2011). Biosynthesis of silver nanoparticles using Mangosteen leaf extract and evaluation of their antimicrobial activities. J. Saudi Chem. Soc..

[13] Patra J.K., Baek K.H. (2016). Green synthesis of silver chloride nanoparticles using *Prunus persica* L. outer peel extract and investigation of antibacterial, anticandidal, antioxidant potential. Green Chem. Lett. Rev..

[14] Yamanaka M., Hara K., Kudo J. (2005). Bactericidal actions of a silver ion solution on *Escherichia coli*, studied by energy-filtering transmission electron microscopy and proteomic analysis. Appl. Environ. Microbiol..

[15] Aritonang H.F., Koleangan H., Wuntu A.D. (2019). Synthesis of silver nanoparticles using aqueous extract of medicinal plants’ (*Impatiens balsamina* and *Lantana camara*) fresh leaves and analysis of antimicrobial activity. Int. J. Microbiol..

[16] Fahmy H.M., Mosleh A.M., Elghany A.A., Shams-Eldin E., Abu Serea E.S., Ali S.A., Shalan A.E. (2019). Coated silver nanoparticles: Synthesis, cytotoxicity, and optical properties. RSC Adv..

[17] Liu L.L., Yang J.L., Shi Y.P. (2010). Phytochemicals and biological activities of *Pulicaria* Species. Chem. Biodivers..

[18] Nematollahi F., Rustaiyan A., Larijani K., Nadimi M., Masoudi S. (2006). Essential oil composition of *Artemisia biennis* willd. and *Pulicaria undulata* (L.) C.A. mey., two compositae herbs growing wild in Iran. J. Essent. Oil Res..

[19] Hegazy M.E.F., Matsuda H., Nakamura S., Yabe M., Matsumoto T., Yoshikawa M. (2012). Sesquiterpenes from an Egyptian herbal medicine, *Pulicaria undulata*, with inhibitory effects on nitric oxide production in RAW264.7 macrophage cells. Chem. Pharm. Bull..

[20] Ali N.A., Jülich W.D., Kusnick C., Lindequist U. (2001). Screening of Yemeni medicinal plants for antibacterial and cytotoxic activities. J. Ethnopharmacol..

[21] El-Kamali H.H., Yousif M.O., Ahmed O.I., Sabir S.S. (2009). Phytochemical analysis of the essential oil from aerial parts of *Pulicaria undulata* (L.) Kostel from Sudan. Ethnobot. Leaflets.

[22] Foudah A.I., Alam A., Soliman G.A., Salkini M.A., Ahmed E.O.I., Yusufoglu H.S. (2016). Pharmacognostical, antioxidant and antimicrobial studies of aerial part of *Pulicaria somalensis* (Family: Asteraceae). Asian J. Biol. Sci..

[23] Ali N.A.A., Sharopov F.S., Alhaj M., Hill G.M., Porzel A., Arnold N., Setzer W.N., Schmidt J., Wessjohann L. (2012). Chemical composition and biological activity of essential oil from *Pulicaria undulata* from Yemen. Nat. Prod. Commun..

[24] Das G., Patra J.K., Debnath T., Ansari A., Shin H.S. (2019). Investigation of antioxidant, antibacterial, antidiabetic, and cytotoxicity potential of silver nanoparticles synthesized using the outer peel extract of *Ananas comosus* (L.). PLoS ONE.

[25] Bagherzade G., Tavakoli M.M., Namaei M.H. (2017). Green synthesis of silver nanoparticles using aqueous extract of saffron (*Crocus sativus* L.) wastages and its antibacterial activity against six bacteria. Asian Pac. J. Trop. Biomed..

[26] National Committee for Clinical Laboratory Standards (NCCLS) (2001). Performance Standards for Antimicrobial Susceptibility Testing: Eleventh Informational Supplement.

[27] Clinical and Laboratory Standards Institute (CLSI) (2012). Reference Method for Dilution Antimicrobial Susceptibility Tests for Bacteria That Grow Aerobically, Approved Standard M7-A6.

[28] Brand-Williams W., Cuvelier M.E., Berset C. (1995). Use of a free radical method to evaluate antioxidant activity. LWT Food Sci. Technol..

[29] Sharifi-Rad M., Salehi B., Sharifi-Rad J., Setzer W.N., Iriti M. (2018). *Pulicaria vulgaris* Gaertn. essential oil: An alternative or complementary treatment for Leishmaniasis. Cell. Mol. Biol..

[30] Vilamová Z., Konvičková Z., Mikeš P., Holišová V., Mančík P., Dobročka E., Kratošová G., Seidlerová J. (2019). Ag-AgCl nanoparticles fixation on electrospun PVA fibres: Technological concept and progress. Sci. Rep..

[31] Niraimathi K.L., Sudha V., Lavanya R., Brindha P. (2013). Biosynthesis of silver nanoparticles using *Alternanthera sessilis* (Linn.) extract and their antimicrobial, antioxidant activities. Coll. Surf. B Biointerfaces.

[32] Marslin G., Siram K., Maqbool Q., Selvakesavan R.K., Kruszka D., Kachlicki P., Franklin G. (2018). Secondary Metabolites in the Green Synthesis of Metallic Nanoparticles. Materials.

[33] Ahmad A., Mukherjee P., Senapati S., Mandal D., Khan M.I., Kumar R. (2003). Extracellular biosynthesis of silver nanoparticles using the fungus *Fusarium oxysporum*. Colloids Surf. B Biointerfaces.

[34] Dhanavath K.N., Islam M.S., Bankupalli S., Bhargava S.K., Shah K., Parthasarathy R. (2017). Experimental investigations on the effect of pyrolytic bio–oil during the liquefaction of Karanja Press Seed Cake. J. Environ. Chem. Eng..

[35] Gopinath V., Priyadarshini S., Priyadharsshini N.M., Pandian K., Velusamy P. (2013). Biogenic synthesis of antibacterial silver chloride nanoparticles using leaf extracts of *Cissus quadrangularis* Linn. Mater. Lett..

[36] Pal S., Tak Y.K., Song J.M. (2007). Does the antibacterial activity of silver nanoparticles depend on the shape of the nanoparticles?. Appl. Environ. Microbiol..

[37] Krishnaraj C., Jagan E.G., Rajasekar S., Selvakumar P., Kalaichelvan P.T., Mohan N. (2010). Synthesis of silver nanoparticles using *Acalypha indica* leaf extracts and its antibacterial activity against water borne pathogens. Colloids Surf. B. Biointerf..

[38] Patil S.V., Borase H.P., Patil C.D., Salunke B.K. (2012). Biosynthesis of silver nanoparticles using latex from few euphorbian plants and their antimicrobial potential. Appl. Biochem. Biotechnol..

[39] Jin J.C., Wu X.J., Xu J., Wang B.B., Jiang F.L., Liu Y. (2017). Ultra small silver nanoclusters: Highly efficient antibacterial activity and their mechanisms. Biomater. Sci..

[40] Ouay B.L., Stellacci F. (2015). Antibacterial activity of silver nanoparticles: A surface science insight. Nanotoday.

[41] Rai A., Prabhune A., Perry C.C. (2010). Antibiotic mediated synthesis of gold nanoparticles with potent antimicrobial activity and their application in antimicrobial coatings. J. Mater. Chem..

[42] Salati S., Doudi M., Madani M. (2018). The biological synthesis of silver nanoparticles by mango plant extract and its anti-Candida effects. J. Appl. Biotechnol. Rep..

[43] Inbathamizh L., Ponnu T.M., Mary E.J. (2013). In vitro evaluation of antioxidant and anticancer potential of *Morinda pubescens* synthesized silver nanoparticles. J. Pharm. Res..

[44] Kumar D.A., Palanichamy V., Roopan S.M. (2015). One step production of AgCl nanoparticles and its antioxidant and photo catalytic activity. Mat. Lett..

[45] Khorrami S., Zarrabi A., Khaleghi M., Danaei M., Mozafari M.R. (2018). Selective cytotoxicity of green synthesized silver nanoparticles against the MCF-7 tumor cell line and their enhanced antioxidant and antimicrobial properties. Int. J. Nanomed..

